# Development of a GC/Q-ToF-MS Method Coupled with Headspace Solid-Phase Microextraction to Evaluate the In Vitro Metabolism of β-Caryophyllene

**DOI:** 10.3390/molecules27217441

**Published:** 2022-11-02

**Authors:** Joseph Lee, Mei Wang, Goutam Mondal, Ikhlas A. Khan, Charles R. Yates

**Affiliations:** 1 National Center for Natural Products Research, School of Pharmacy, University of Mississippi, University, MS 38677, USA; 2 Natural Products Utilization Research Unit, Agricultural Research Service, United States Department of Agriculture, University, MS 38677, USA; 3 Division of Pharmacognosy, Department of BioMolecular Sciences, School of Pharmacy, University of Mississippi, University, MS 38677, USA

**Keywords:** GC/Q-ToF-MS, headspace solid-phase microextraction (HS-SPME), β-caryophyllene, in vitro metabolism

## Abstract

Sample preparation remains both a challenging and time-consuming process in the field of bioanalytical chemistry. Many traditional techniques often require multi-step processes, which can introduce additional errors to the analytical method. Given the complexity of many biological matrices, thorough analyte extraction presents a major challenge to researchers. In the present study, a headspace solid-phase microextraction (HS-SPME) coupled with a GC/Q-ToF-MS method, was developed to quantify in vitro metabolism of β-caryophyllene by both human liver microsome (HLM) and S9 liver fractions. Validation of the method was demonstrated both in terms of linearity (*R*^2^ = 0.9948) and sensitivity with a limit of detection of 3 ng/mL and a limit of quantitation of 10 ng/mL. In addition, the method also demonstrated both inter- and intra-day precision with the relative standard deviation (RSD) being less than 10% with four concentrations ranging from 50–500 ng/mL. Since this method requires no solvents and minimal sample preparation, it provides a rapid and economical alternative to traditional extraction techniques. The method also eliminates the need to remove salts or buffers, which are commonly present in biological matrices. Although this method was developed to quantify in vitro metabolism of one analyte, it could easily be adapted to detect or quantify numerous volatiles and/or semi-volatiles found in biological matrices.

## 1. Introduction

Although many technological advances have been made in the field of bioanalytical chemistry, sample preparation remains both a challenging and time-consuming process [1]. Many techniques often require that the analyte be extracted from the sample matrix in order to be analyzed by instrumentation. Due to the complexity of many biological matrices, thorough analyte extraction can be a major challenge to researchers [1]. Not only does this increase the sample preparation time, but it also introduces the possibility of errors entering the analytical process.

Headspace solid-phase microextraction (HS-SPME) is a rapid, sensitive, solvent-free, and economical method of extracting analytes from a variety of matrices by partitioning them from a liquid or gaseous sample onto an immobilized stationary phase. It uses a very simple setup and requires no additional instrumentation other than a conventional gas chromatograph (GC). HS-SPME was first introduced by Pawliszyn [2] for the evaluation of pollutants in water. Since then, HS-SPME has been applied in a variety of analytical applications with increased popularity. One of the primary advantages of HS-SPME over traditional analytical techniques is the elimination of the need to separate the analyte of interest from biological matrices in order to be analyzed [3]. Moreover, HS-SPME eliminates preconcentration steps by directly extracting the analytes onto a poly-(dimethylsiloxane)-coated fiber. Thus, HS-SPME can be used as an efficient sample extraction technique that can overcome the difficulties in sample preparation [4,5,6].

HS-SPME has routinely been used as an analytical technique for food and plant material [7,8,9]. For example, the analysis of volatile compounds of six Rhododendron species was accomplished using a HS-SPME technique coupled with GC/Q-ToF-MS [9]. However, other HS-SPME applications have also been reported, including the analyses of both biological fluids and matrices [1,3,10]. For instance, Walker and colleagues described using the SPME technique coupled with GC/MS in order to analyze metabolites formed by activated rat liver microsomes in the presence of volatile hydrocarbons [11]. In addition, a small number of human biological fluids (blood and urine) and tissues (brain, lung, and adipose) were analyzed using the same SPME technique [11]. Another study demonstrated the use of SPME coupled with LC/MS to analyze S9 liver fraction metabolism of six polycyclic aromatic hydrocarbons in trout. The authors of this study utilized an immersion SPME technique in order to collect the analyte [12].

Copaiba oil and its major compounds have been the subject of numerous in vitro studies. For example, Lima and colleagues demonstrated that copaiba oil resin exhibited tumoricidal activity against melanoma cells [13]. Another study combined in vitro and in vivo methods in order to demonstrate the anti-inflammatory activity of copaiba oil from three species of Copaifera [14]. In addition to copaiba oil, individual major compounds present in the oil have been investigated. The compounds β-caryophyllene, caryophyllene oxide, and α-humulene were investigated utilizing in vitro methods in order to investigate possible drug metabolism inhibition. Both human and rat liver microsomes were used to demonstrate that the investigated compounds, particularly caryophyllene oxide, possessed the ability to inhibit CYP3A enzyme activities. As a result, these compounds have the potential to cause adverse drug reactions [15]. Although these are but a few examples of in vitro testing of copaiba oil and its major compounds, to the authors’ knowledge, this is the first report concerning the study of in vitro metabolism of β-caryophyllene utilizing a HS-SPME method coupled with GC/Q-ToF-MS.

The primary goal of the present study was to develop and validate a HS-SPME method coupled with GC/Q-ToF-MS in order to quantitate the in vitro metabolism of β-caryophyllene by both human liver microsome (HLM) and S9 liver fractions. Within this study, we report both the optimization and validation of the developed HS-SPME technique and its application in the analysis of metabolites present in samples containing either HLM or S9 biological matrices.

## 2. Results

### 2.1. HS-SPME GC/Q-ToF-MS Method Development and Optimization

**Figure 1:**
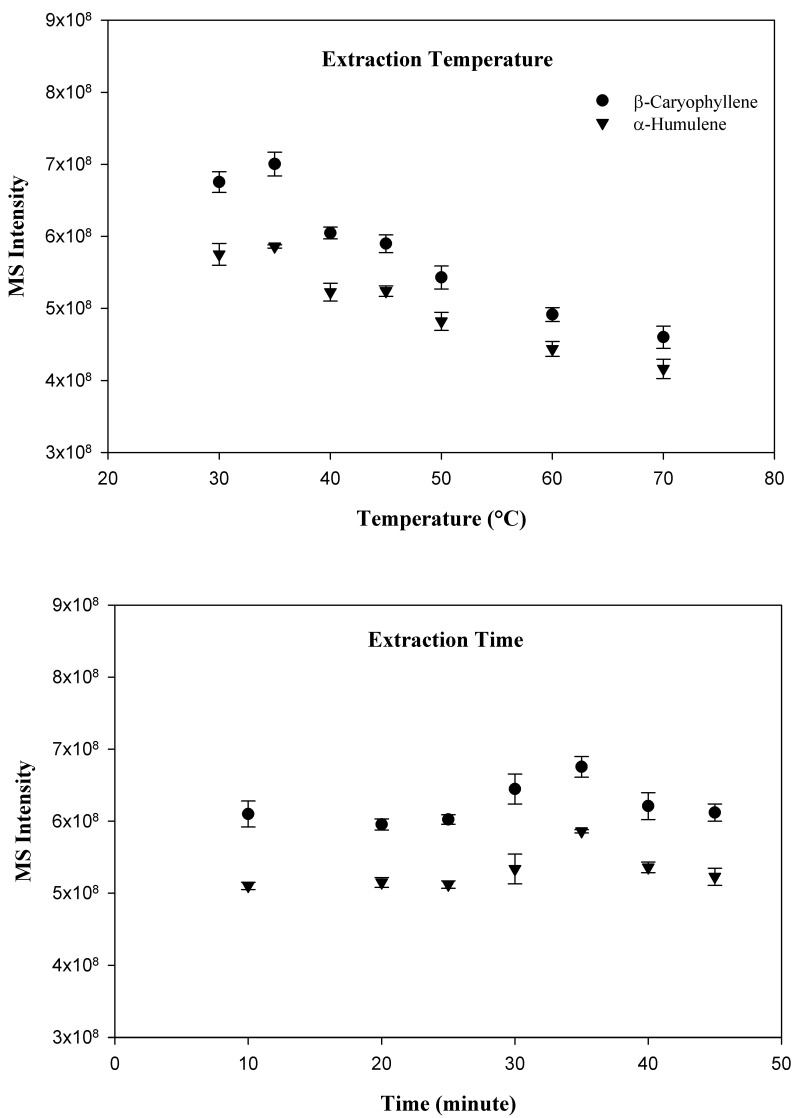
SPME method optimization in terms of extraction temperature and time.

Three different commercially available SPME fibers, including carboxen/PDMS, PDMS, and PDMS/DVB were evaluated, however, the PDMS fiber was selected for the present study because it provided the best peak shape and sensitivity for the targeted compounds. In order to optimize the variables which effect adsorption of compounds to the SPME fiber, a time and temperature study was conducted. Since the SPME technique relies primarily upon the volatility of the investigated compounds, the optimal adsorption temperature was the first parameter to determine. The following sample incubation temperatures were investigated: 30, 35, 40, 45, 50, 60, and 70 °C. Interestingly, the temperature experiment demonstrated that the greatest adsorption of the target compound occurred at the relatively low temperature of 35 °C. As the incubation temperature increased beyond 35 °C, the amount of both compounds adsorbed tended to decline. After the determination of the optimal incubation temperature, a time optimization study was conducted. The following incubation times were investigated: 10, 20, 25, 30, 35, 40, and 45 min. As expected, with an increase in incubation time, a greater amount of target compound and internal standard were adsorbed by the SPME fiber. Adsorption tended to stabilize at 35 min. Beyond 35 min, compound adsorption did not increase. As a result, an incubation time of 35 min at 35 °C was chosen for the investigation. The results for the extraction temperature and time study are shown in Figure 1.

### 2.2. HS-SPME GC/Q-ToF-MS Method Validation

The developed chromatographic method was validated to determine the linearity, range, inter-day, intra-day precision accuracy, limit of detection (LOD) and limit of quantification (LOQ) according to the ICH and FDA guidelines [16,17].

#### 2.2.1. Selectivity

**Figure 2:**
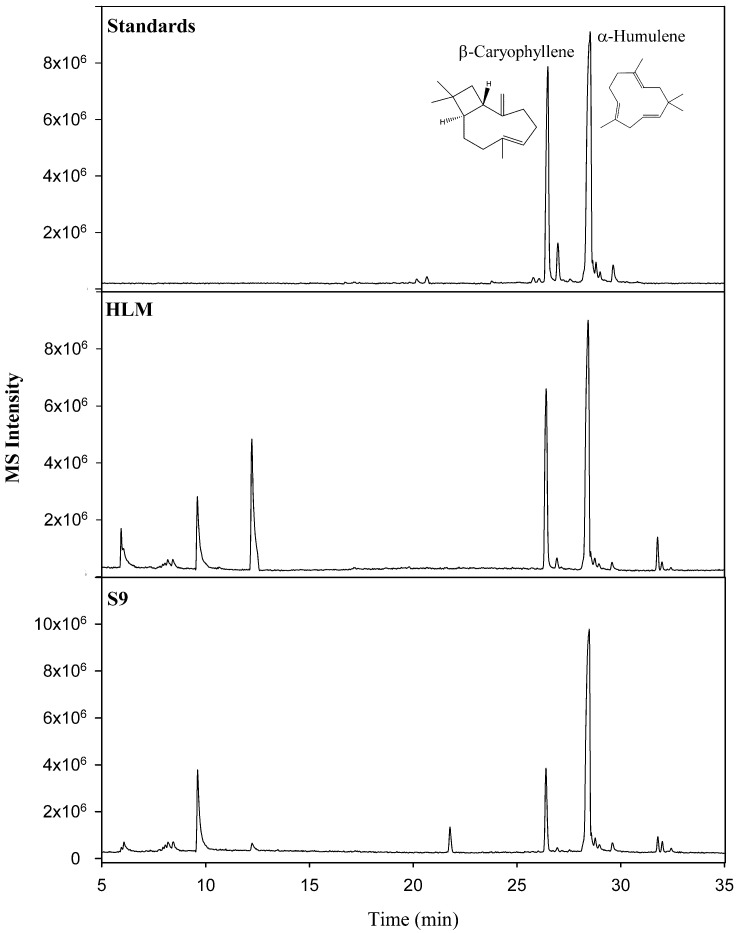
Comparison of representative total ion chromatograms of the chemical standards, human liver microsome (HLM), and S9 liver fractions.

Although similar in structure and molecular weight, good chromatographic separation was achieved both by column selection and GC oven temperature programming. As a result, peaks could be easily identified as β-caryophyllene (*R_t_*: 26.46 min) and α-humulene (*R_t_*: 28.53 min, internal standard) and confirmed with the high-resolution accurate mass spectral data and reference standards. A comparison of the representative total ion chromatograms of the chemical standards, HLM, and S9 liver fractions are shown in Figure 2. In order for the quantitative analysis software to perform calculations, quantifier ions must be selected for both the compound of interest (β-caryophyllene) and the internal standard (α-humulene). As a result, the quantifier ions of *m/z* 91.0780 and *m/z* 93.0939 were selected to represent β-caryophyllene and α-humulene, respectively. Given the good chromatographic separation of the target and internal standard, the quantitative software could easily identify both compounds.

#### 2.2.2. Linearity and Sensitivity

With the data obtained from the standards, a calibration curve was constructed utilizing a second order equation. A *R*^2^ value of 0.9948 demonstrated a good calibration curve within the concentration range 10–1000 ng/mL. The LOD and LOQ were determined to be 3 ng and 10 ng by signal-to noise ratios of 3:1 and 10:1, respectively. 

#### 2.2.3. Precision and Accuracy

Intra- and inter-day precision and accuracy of the developed SPME GC/Q-ToF-MS method were evaluated by analyzing QC samples at 50, 75, 250, and 500 ng/mL, on a single day and on three different days. Both precision and accuracy were well within the 20% acceptance range [17]. The relative standard derivation (RSD, %) for intra- and inter-day precision were below 10%. The detailed information is given in Table 1 and Table 2.

### 2.3. In Vitro Metabolism

**Figure 3:**
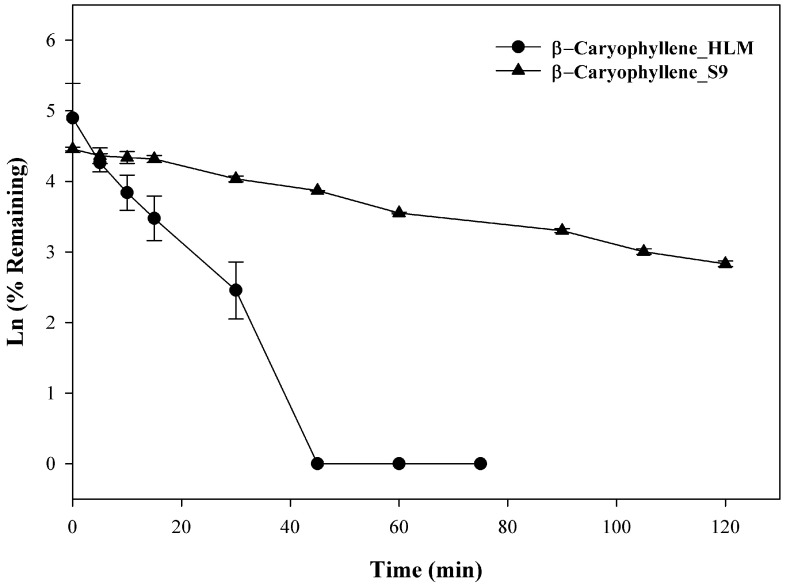
Time-dependent metabolic depletion of β-caryophyllene in pooled human liver microsome and human S9 fractions. Human liver microsomes and human S9 fractions were incubated with β-caryophyllene (10 µM) for 0–75 min and 0–120 min in presence of cofactors, respectively. The data are expressed as mean ± SEM (*n* = 3).

Previously, 14-hydroxycaryophyllene was isolated from rabbit urine following oral administration of β-caryophyllene and was subsequently shown to derive from the intermediate metabolite β-caryophyllene oxide [18]. No further work has been done to identify specific metabolic pathways, however, it is reasonable to speculate that liver-mediated Phase I metabolism was involved. In the present investigation, β-caryophyllene (10 µM) was incubated with liver microsomes up to 120 min, the rate of clearance, a T_1/2_ of 9.6 ± 0.1 min with a CL′int of 194.9 ± 1.1 mL/min/kg was observed in HLM, whereas with S9 fraction that utilizes UDPGA as cofactors, a T_1/2_ of 44.4 ± 0.9 min and a CLint of 42.2 ± 0.8 mL/min/kg were observed. As seen in Figure 3, β-caryophyllene disappeared rapidly and showed extensive metabolic depletion at 30 min (~95% depleted) in HLM, whereas ~44% depletion was seen with S9 fractions at the same time. At the end of the incubation (120 min) ~14% of the compound was found unchanged with S9 fractions while no compound was found from 45–75 min in HLM fraction. The observation demonstrated direct evidence that β-caryophyllene was extensively metabolized via both Phase I and Phase II pathways. This high metabolic instability may explain β-caryophyllene’s low oral bioavailability observed previously in rats [19]. Studies to determine the exact structure of said metabolites were beyond the scope of the current investigation, but are the focus of future studies.

## 3. Materials and Methods

### 3.1. Materials

Human liver microsomes and S9 fractions (pooled mixed sex) were procured from In Vitro Technologies Inc. (Melbourne, Australia). G-6-PDH, glucose-6-phosphate, NADP+, and UDPGA were procured from Sigma Chemical Company (St. Louis, MO, USA). In addition, β-caryophyllene and α-humulene were also procured from Sigma Chem. Co., with the purity of both compounds being confirmed by chromatographic analysis to be ≥95%. All other chemicals were procured from Fisher Scientific (Pittsburgh, PA, USA). Headspace vials (20 mL) were purchased from Agilent Technologies (Santa Clara, CA, USA). Polydimethylsiloxane (PDMS) (100 µm × 23 Ga), carboxen/PDMS (85 µm × 23 Ga), and PDMS/divinylbenzene (65 µm × 23 Ga) SPME fibers were procured from Supelco (Bellefonte, PA, USA).

### 3.2. Sample Preparation

All samples were prepared in 20 mL headspace vials. Sample preparation involved pipetting 2 mL HPLC grade water into the vial, followed by the addition of 200 µL of saturated sodium chloride solution. Next, the biological sample (500 µL) was vortexed and pipetted into the vial. This mixture was then placed upon the SPME auto-sampler in order to be analyzed using the GC/Q-ToF-MS. This process was repeated for each additional sample.

### 3.3. HS-SPME and GC/Q-TOF-MS Instrument Conditions

Analysis of all prepared samples was performed utilizing an Agilent 7890B gas chromatographic (GC) instrument which was equipped with a RS185 PAL3 autosampler. The GC was connected to an Agilent 7250 Accurate-Mass Quadrupole Time-of-Flight (Q-ToF) mass spectrometer. The capillary column (30 m × 0.25 mm i.d.) utilized was coated with a 5% phenyl methyl siloxane (Agilent J&W HP-5MS) film (0.25 µm). Helium at a constant flow rate of 1 mL/min was used as the carrier gas. Each sample was analyzed using the following GC oven program: 70 °C held for 1 min, then heated at a rate of 2 °C/min to 155 °C, and finally heated at a rate of 10 °C/min to 250 °C. A post run time of 5 min at 280 °C was also utilized to ensure that no sample crossover occurred. The inlet was programmed at 280 °C in splitless mode, while the SPME fiber was desorbed for 1 min in the inlet. The transfer tube from the GC to the Q-ToF-MS was held at 280 °C throughout the experiment.

Prior to sample desorption in the GC inlet, a Supelco #57341-U SPME fiber (100 µm PDMS × 23 Ga, Bellefonte, PA, USA) was conditioned for 5 min at 260 °C. Each sample was incubated for 5 min at 35 °C with agitation. After 5 min, the SPME fiber was inserted into the vial and allowed to adsorb sample for 35 min at 35 °C with agitation. After the SPME fiber was desorbed in the GC inlet for 1 min, the SPME fiber was conditioned for 5 min at 260 °C in order to prepare for the next sample injection.

The Q-ToF mass spectrometer was equipped with a high-emission low-energy electron ionization source which was operated with an electron energy of 70 eV and an emission current of 5.0 µA. The source, quadrupole, and transfer line temperatures were 230 °C, 150 °C, and 280 °C, respectively during the experiment. All mass spectra data were recorded at a rate of 5 Hz from *m/z* 35 to 450 after a 4 min solvent delay. Data was acquired utilizing Agilent MassHunter software (version B7.06.274), while quantification was achieved with Agilent MassHunter Quantitative Analysis (version 10.0.707.0, Santa Clara, CA, USA). The NIST database (version 2.3, The National Institute of Standards and Technology, Gaithersburg, MD, USA ) was utilized for tentative compound identification. All compounds were later confirmed with analytical standards.

### 3.4. Quantitative Analysis 

In order to establish a calibration curve, a series of standard solutions was prepared in methanol. The following standard concentrations for β-caryophyllene were prepared: 10, 25, 50, 75, 100, 175, 250, 375, 500, 750, and 1000 ng/mL. In addition to the target compound, each calibration standard contained a final concentration of 500 ng/mL of the internal standard (α-humulene). Triplicates of each calibration standard were prepared utilizing the sample preparation procedure previously mentioned.

### 3.5. Method Validation 

The developed HS-SPME GC/Q-ToF-MS method was validated by determining the linearity, intra-day and inter-day precision, accuracy, limit of detection (LOD), and limit of quantification (LOQ) according to ICH guidelines [16].

### 3.6. Metabolic Stability of β-Caryophyllene in Human Liver Microsomes and S9 Fractions

Phase I and Phase II metabolic stability of β-caryophyllene was determined using human liver microsomes and S9 fractions using methods previously described [20]. Following reaction mixture priming (5 min at 37 °C), β-caryophyllene (10 μM) was added. Reaction mixture aliquots (300 μL) were collected at predetermined time points (0, 10, 20, 30, 45, 60, 90, and 120 min) followed by extraction with ice-cold acetonitrile/methanol (50:50, 600 μL) containing the internal standard α-humulene (500 ng/mL). Subsequent to centrifuging (15 min at 12,000 RPM 4 °C), supernatants were transferred to GC/Q-ToF-MS for analysis. Half-life (T_1/2_) and intrinsic clearance (CLint, mL/min/kg) were calculated as:

T_1/2_ = 0.693/k

where the slope (k) of the line obtained by plotting Ln% of remaining β-caryophyllene versus incubation time. CLint = (0.693/in vitro T_1/2_) × (mL incubations/mg microsomes) × (45 mg microsomes/gm liver) × (20 gm liver/kg BW).

## 4. Conclusions

The extraction of analytes from sample matrices often requires multiple steps which add both material and labor cost, as well as introduce the potential for errors. With the use of HS-SPME, the extraction process can be eliminated. Although HS-SPME does not totally eliminate the need for sample preparation prior to analysis, this step is greatly simplified when compared to traditional extraction methods. With the development and validation of the described HS-SPME method, we were able to successfully quantify a target compound present in a biological matrix without the need for extraction. By using a series of optimization trials, the analyte equilibrium between the sample matrix and SPME fiber was found to be best achieved with an incubation temperature of 35 °C and an extraction time of 35 min. This simple, yet robust method not only provided results for the present in vitro study, but it also demonstrated that it could be adapted for use in future in vitro, and perhaps in vivo studies which involve analytes within complex biological matrices. Overall, it is the hope of the authors that this method will provide great utility in the future for a wide variety of projects.

## Figures and Tables

**Table 1:** Intra-day assays of samples.

Compound	Intra-Day									
	Nominal Conc. (ng/mL)	Day-1 (*n* = 3)			Day-2 (*n* = 3)			Day-3 (*n* = 3)		
		Detected Conc. (ng/mL)	Precision (RSD%)	Accuracy (%)	Detected Conc. (ng/mL)	Precision (RSD%)	Accuracy (%)	Detected Conc. (ng/mL)	Precision (RSD%)	Accuracy (%)
**β-caryophyllene**	50	50.09	1.23	100.17	52.38	3.44	104.76	50.83	2.64	101.67
	75	81.98	2.81	109.66	82.24	4.43	109.66	80.34	5.20	107.12
	250	281.92	6.91	112.77	248.60	3.48	99.44	283.18	2.63	113.27
	500	490.78	4.90	98.16	530.53	3.18	106.11	534.53	2.90	106.91

**Table 2:** Inter-day assays of samples.

Compound	Nominal Conc. (ng/mL)	Inter-Day (*n* = 9)		
		Detected Conc. (ng/mL)	Precision (RSD%)	Accuracy (%)
β-**caryophyllene**	50	51.10	4.16	102.20
	75	81.52	3.84	108.70
	250	271.23	7.52	108.49
	500	518.62	5.15	103.72

## Data Availability

Not applicable.
